# Acute Toxicity of an Emerging Insecticide Pymetrozine to *Procambarus clarkii* Associated with Rice-Crayfish Culture (RCIS)

**DOI:** 10.3390/ijerph15050984

**Published:** 2018-05-14

**Authors:** Jixin Yu, Elvis Genbo Xu, Wei Li, Shiyu Jin, Ting Yuan, Jiashou Liu, Zhongjie Li, Tanglin Zhang

**Affiliations:** 1 State Key Laboratory of Freshwater Ecology and Biotechnology, Institute of Hydrobiology, Chinese Academy of Sciences, 7 South Donghu Road, Wuhan 430072, Hubei, China; jxyu001@126.com (J.Y.); liwei@ihb.ac.cn (W.L.); jinshiyu@ihb.ac.cn (S.J.); yuanting@ihb.ac.cn (T.Y.); jsliu@ihb.ac.cn (J.L.); zhongjie@ihb.ac.cn (Z.L.); 2 College of Life Sciences, University of Chinese Academy of Sciences, Beijing 100049, China; 3 Department of Chemical Engineering, McGill University, Montreal, QC H3A 0C5, Canada; genbo.xu@mail.mcgill.ca

**Keywords:** pesticide, aquatic toxicology, behavioral effects, histopathology, freshwater crayfish

## Abstract

This study aims to evaluate the acute toxicity of pymetrozine to juvenile *Procambarus clarkii*. Two 96-h toxicity tests were conducted to assess the lethal concentration 50 (LC_50_) values, behaviors, and histopathology (at 50% of the 96 h LC_50_) after pymetrozine exposure. The results showed high toxicity of pymetrozine to juvenile *P. clarkii* in a dose and time dependent manner, with a decreasing LC_50_ from 1.034 mg/L at 24 h to 0.479 mg/L at 96 h. The maximum allowable concentration (MAC) of pymetrozine for *P. clarkii* was 0.106 mg/L. Behavioral abnormalities were observed in pymetrozine-treated crayfish, such as incunabular hyperexcitability, subsequent disequilibrium, lethargy, and increased defecation. Significant lesions were observed in all pymetrozine-treated tissues, including: (1) in gill, hemocytic infiltration and 33.27% of epithelial cells lesions; (2) in perigastric organs, 64.37%, 29.06%, and 13.99% of tubules with lumen atrophy, vacuolation, and cell lysis, respectively; (3) in heart, 2.5%, 8.55% and 7.74% of hemocytic infiltration, vacuolization, and hyperplasia, respectively; (4) in stomach, 80.82%, 17.77%, 6.98%, 5.24% of cuticula swelling, vacuolization, muscle fragmentation, hemocytic infiltration, respectively; (5) in midgut, 7.45%, 10.98%, 6.74%, and 13.6% of hyperplasia, tissue lysis and vacuolation, hemocytic infiltration, muscle fracture; and (6) in abdominal muscle, 14.09% of myofiber fracture and lysis. This research demonstrates that pymetrozine is highly toxic to juvenile *P. clarkii*, with significant effects on mortality, behavior and histopathology at concentrations of ≤1.1 mg/L, while the estimated practical concentration of pymetrozine in rice-crayfish culture water was around 20 times lower than the calculated MAC.

## 1. Introduction

The red swamp crayfish, *Procambarus clarkii*, is a widely-distributed freshwater crayfish and key species in many water bodies, and has great influences on the ecosystems, such as water quality, sediment, food web, and biodiversity [1,2,3]. *P. clarkii* has been widely used in water quality and pollution determination as a model organism [4,5,6].

*P. clarkii* has also become a globally important cultured species in crayfish industry, with an annual yield of ~9 × 10^5^ t and the highest share in global freshwater crayfish [7,8]. In major culturing regions, such as China, the USA and Portugal, crayfish culture is commonly combined with rice planting as rice-crayfish culture (RCIS) [9,10]. Pesticides used in RCIS, however, may affect the health and production of crayfish. Due to its ecological and commercial values as human food, determination of the toxicity of pesticide to *P. clarkii* is significant for pesticide application in RCIS.

Many pyrethroids (e.g., cypermethrin, cyfluthrin, deltamethrin, lambda-cyhalothrin, etofenprox), organophosphorus pesticides (e.g., chlorpyrifos, thiobencarb), and organochlorines (e.g., chlorantraniliprole) [2,4,10,11] have been proved to be highly toxic to *P. clarkii*, with the 96 h lethal concentration 50 (LC_50_) values lower than 0.1 mg/L. Misuse of pyrethroids [10] and fipronil [12] has been reported to cause severe crayfish kills in RCIS. The non-target crayfish in rice fields may be poisoned by rice pesticide application, and in other water bodies by pesticide through drainage, migration of spray, surface runoff and even food chain [6,10].

Pymetrozine, 6-methyl-4-[(E)-(pyridin-3-ylmethylene) amino]-4,5-dihydro-2H-[1-3]-triazin-3 one, is a pyridine azomethine compound, which represents an emerging insecticide for plant-sucking insects, such as aphids, whiteflies and plant hoppers [13]. It has been widely applied on rice and other crops in China as a substitute of organophosphorus pesticides since approximately 2006 [13], and rated as a Class 1 pesticide (i.e., harmless according to the International Organization for Biological and Integrated Control Scale) [14]. Previous studies showed its relatively low toxicity to many aquatic and ricefield-relative organisms (Table 1). However, a primary study showed that pymetrozine at a concentration of 1.0 mg/L caused 100% mortality of shrimp *Penaeus vannamei* [15], which is highly toxic. Large crustacea are known to share certain physiological similarities with insects [16]. Pymetrozine is thus probably to be highly toxic to *P. clarkii*. Additionally, field study indicated a pymetrozine concentration of 0.305 mg/L in water after 2 h with a dosage of 600 g a.i./ha [13], which is potentially to be lethal to *P. clarkii*. Thus, it is necessary to determine the toxicity of pymetrozine to the non-target crayfish *P. clarkii*.

In this study, two 96 h toxicity tests were performed to assess the lethal (mortality) and sublethal effects (behavior and histopathology in gill, perigastric organ, heart, stomach, midgut, and abdominal muscle) of pymetrozine on juvenile *P. clarkii*. The LC_50_ values at 24, 48, 72 and 96 h were obtained from the first 96 h experiment, along with the behavioral changes and maximum allowable concentration (MAC), and the histopathological alterations were determined at a sublethal concentration of pymetrozine in the second 96 h experiment. Furthermore, a safety evaluation of pymetrozine was conducted to estimate the ecological risk of applying pymetrozine in RCIS. The results will provide a better understanding of the toxicity of pymetrozine to aquatic animals, and a guideline for pymetrozine application in RCIS.

## 2. Materials and Methods

### 2.1. Ethical Statement

All procedures performed in studies involving animals were in accordance with ethical standards in Laboratory animal—Guideline for Ethical Review of Animal Welfare (The National Standard of the People’s Republic of China GB/T 35892-2018). All dissections were performed under MS-222 anesthesia. In addition, all efforts were made to minimize suffering.

### 2.2. Test Organisms and Chemical

Juvenile *P. clarkii* were supplied by a crayfish breed cooperative in Qianjiang City, Hubei Province, China. Crayfish were transported to the laboratory of Institute of Hydrobiology, Chinese Academy of Sciences, and acclimated for 14 days, according to Yu et al. [6]. Mortality during acclimation was below 5%. Healthy intermolt-staged crayfish (mean weight of 0.27 ± 0.05 g) with complete appendages were selected.

Technical-grade pymetrozine (purity 99.10%, Beijing JSYH Chemical Technology Research Institute, Beijing, China) was dissolved in double-distilled water as a 0.1 g/L stock solution.

### 2.3. Test Conditions

Tap water was used in exposure tank after a 48-h aeration for chlorine elimination and ultraviolet sterilization. Water temperature and photoperiod were maintained at 20 °C and a 16:8 h light:dark cycle, respectively. Water quality were daily measured, and the dissolved oxygen, hardness (CaCO_3_), ammonia, water temperature, and pH were 5.7 ± 0.8, 127 ± 9, <0.1 mg/L, 7.47–7.86, and 20 ± 0.6 °C, respectively.

### 2.4. Acute Toxicity Tests

A 96-h semi-static bioassay was conducted with a daily renewal of pymetrozine solution to maintain the test concentrations. No feeding was conducted during the 96-h exposure, and artificial *Elodea nuttallii* and PVC pipes were provided in the exposure tanks to minimize aggression and cannibalism of the crayfish. A preliminary range-finding test (from 0.01 to 100 mg/L) was conducted and then six concentrations (0.1, 0.3, 0.5, 0.7, 0.9, 1.1 mg/L at nominal) were chosen for the following exposure. Ten crayfish per triplicated tank were exposed to 10 L pymetrozine solution of each concentration or control water (*N* = 21). Behavioral changes and mortality of the crayfish were recorded at 1, 12, 24, 48, 72 and 96 h after the introduction. Death was defined as lack of any movement of a crayfish within 5 min when probed gently with a glass rod, and dead crayfish were removed from the tanks [6].

### 2.5. Histopathology Test

Ten crayfish per triplicated tank were exposed to nominal 0.24 mg/L pymetrozine (50% of the 96 h LC_50_) or control water (*N* = 6). At 96 h, gills, perigastric organs, hearts, stomachs, midguts and abdominal muscles of 12 survivors were freshly dissected out, separately. The tissues were fixed in Bouin’s Solution, dehydrated in a graded series of ethanol, cleared in xylene, and then embedded in paraffin wax. Sections of 4 μm were prepared and stained with hematoxylin-eosin (H&E) [22]. An OLYMPUS BX53 microscope (Olympus Corporation, Tokyo, Japan) was used to examine the sections, and the histological impacts were quantified by measuring the percentages of different lesions’ area, number or length in three sections as repeats (*N* = 3) [6].

### 2.6. Statistical Analysis

The mortalities at 24, 48, 72 and 96 h were used to determine the 24, 48, 72 and 96 h LC_50_ and 95% confidence with Probit analysis [16] in SPSS 13.0 (IBM, Armonk City, NY, USA). The MAC of pymetrozine in water was calculated by Reed-Muench method [23]. Mortalities in the regressions and percentages of lesions were given as mean ± standard error (Mean ± SE). Analysis of the quantitative histology was performed in SPSS 13.0, and normality of percentages or transformed percentages of the lesions were tested. Data on hemocytic infiltration in stomach were analyzed using Mann-Whitney *U* test and independent-sample *t*-test for the rest. A probability of *p* < 0.05 was considered to be significant.

## 3. Results

### 3.1. Mortality, LC_50_ Values and MAC

**Figure 1:**
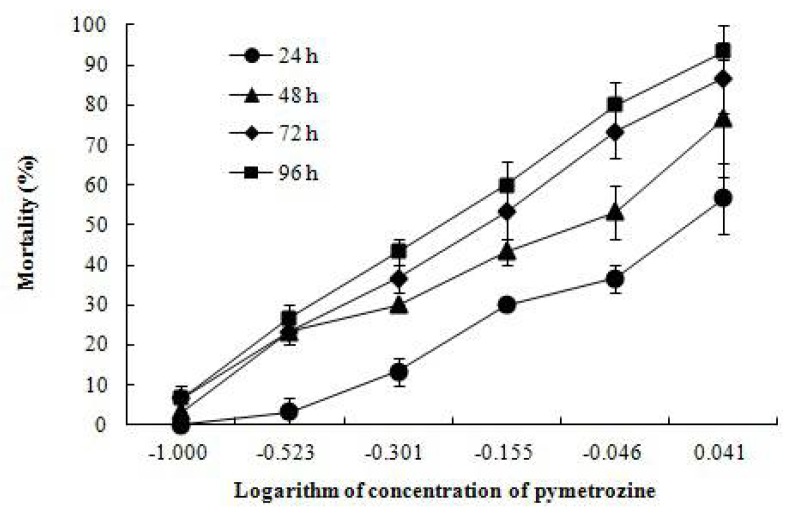
Mortality of pymetrozine-exposed juvenile *P. clarkii* at 24, 48, 72 and 96 h (Mean ± SE).

The mortalities of crayfish increased with time and increasing pymetrozine concentrations (Figure 1). No mortality was observed in control tanks. The 24, 48, 72 and 96 h LC_50_ were 1.034, 0.724, 0.551 and 0.479 mg/L, respectively (Table 2). The calculated MAC of pymetrozine in water was 0.106 mg/L.

### 3.2. Behavioral Responses

Behavioral abnormalities were only found in the pymetrozine-exposed crayfish. The initial response to pymetrozine was hyperexcitability, such as fast movement, climbing the chamber wall, or increased agonism. Then, some individuals showed body jerk or belly arch, and then slow movement, equilibrium loss, sank to the bottom, and lethargy. Increased defecation was noted in pymetrozine-exposed crayfish compared with controls.

### 3.3. Histopathological Effects of Pymetrozine

#### 3.3.1. Gills

**Figure 2:**
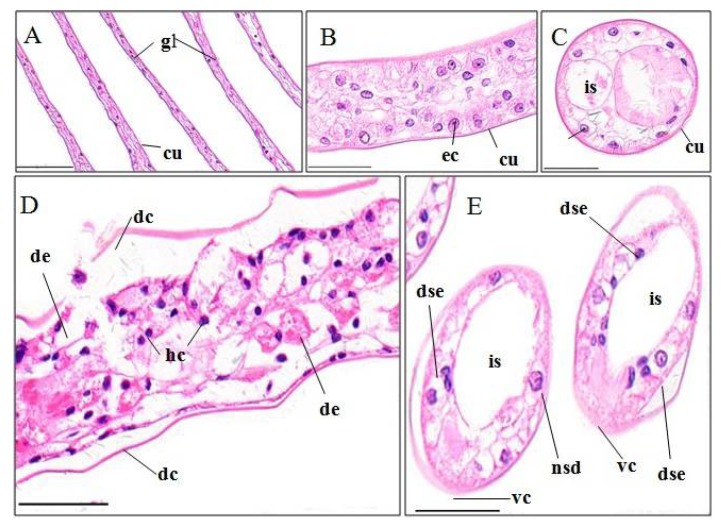
Gill structure of *P. clarkii* following 96 h exposure to a control solution (**A**–**C**) and 0.24 mg/L pymetrozine (**D**,**E**). Control gill with clear and uniform gill lamella (gl) and cuticula (cu) ((**A**), 200×), uniform arrangement of epithelial cells (ec) ((**B**), 400×) and uniform intralamellar space (is) ((**C**), 400×). Damaged gill lamella showing granular hemocytes (hc) inside the intralamellar space, degeneration and vagueness of the cuticula (dc, vc), epithelial cell lysis and disorganization (de), nucleus swelling and darkening (nsd), and separation from the cuticula (dse) ((**D,E**), 400×). cu, cuticula; ec, epithelial cell; is, intralamellar space. H&E stain, scale bars = 100 μm.

**Figure 3:**
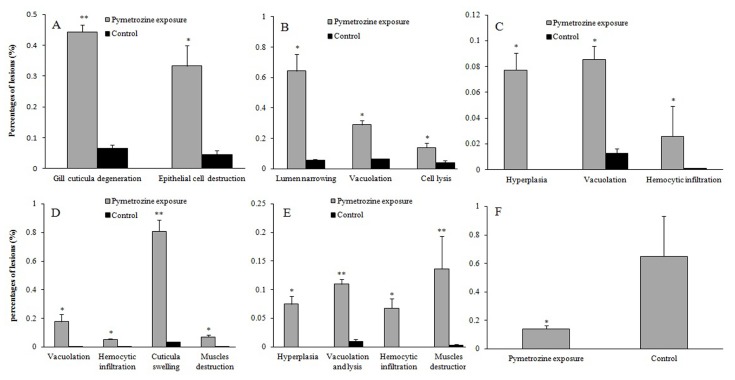
Percentages of number, area or length of lesions in gill (**A**), perigastric organ (**B**), heart (**C**), stomach (**D**), midgut (**E**) and abdominal muscle (**F**) of the pymetrozine-exposeed and control *P. clarkii* (Mean ± SE). All of the rounded and elliptical gill crosscuts, and tubules of the perigastric organs were quantified by counting the total number and those with different lesions; whole the stomach cuticula and the cuticula with swelling were measured on length, and other lesions in stomach, all the lesions in hearts, midguts and abdominal muscles were quantified by measuring areas of the total samples and parts with different lesions, separately. Percentage of hemocytic infiltration in stomach was analyzed using Mann-Whitney *U* test, and others were analyzed using independent-sample *t-*test. Asterisks indicate significant differences between the control and treatment groups (* *p* < 0.05, ** *p* < 0.01).

Gills of *P. clarkii* is composed of branching gill filaments (lamellae) (Figure 2A), which is covered by a thick cuticula underlain with a single epithelial layer (Figure 2B). The control gills showed uniform arrangements of lamellae and intralamellar spaces, clear cuticula (Figure 2A–C), and closely and uniformly located epithelial cells (Figure 2B,C). A significantly higher percentage of gill cuticula vagueness and degeneration (44.29 ± 2.21%) was observed after the pymetrozine exposure than in controls (6.55 ± 1.09%) (Figure 2D,E; *p* < 0.001, Figure 3A). A significantly higher percentage of gill epithelial cells exhibited lesions (e.g., cell disorganization and detachment from the cuticula) in pymetrozine-treated groups (33.27 ± 6.65%) than the control group (4.58 ± 1.34%) (Figure 2E; *p* < 0.05, Figure 3A). Hemocytic infiltration in the intralamellar space was also found in the pymetrozine-exposed gills (Figure 2D).

#### 3.3.2. Perigastric Organs

**Figure 4:**
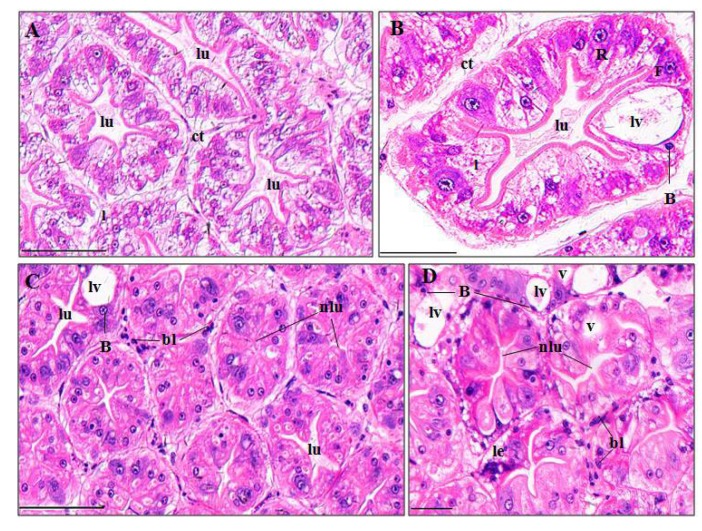
Perigastric organ structure of *P. clarkii* following 96 h exposure to a control solution (**A**,**B**) and 0.24 mg/L pymetrozine (**C**,**D**). Control perigastric organs with intact and tight tubules and intertubular connective tissues (ct) ((**A**), 200×); control tubules with intact and recognizable epithelial cells and stellate lumina. Note the large vacuole (lv) which occupies most of the B-cell ((**B**), 400×). Damaged perigastric organs showing narrowing of the lumina (nlu) ((**C**), 200×; (**D**), 400×), vacuolation (v), cell lysis (le) of tubules (**D**), some black granules in the connective tissues (bl) (**C,D**), and decreased lipid granules in the tubules (**C,D**). lu, lumen; ct, connective tissue; B, B-cell; F, F-cell; R, R-cell; l, lipid granules. H&E stain, scale bars = 100 μm.

Perigastric organs of *P. clarkii* consist of blind ended tubules which are bound by connective tissues (Figure 4A). The control perigastric organs showed unbroken and uniform tubules (Figure 4A), and the tubules were tight, unwounded and recognizable as three types of epithelial cell (absorptive (R) cell, secretory (B) cell and fibrillar (F) cell) and centrally located stellate lumina (Figure 4B). In the pymetrozine-treated perigastric organs, 64.37 ± 10.99%, 29.06 ± 2.51%, 13.99 ± 3.06% of tubules exhibited apparent narrowing of lumen (Figure 4C), vacuolation, and cell lysis (Figure 4D), respectively, which were significantly higher than those of the control group (*p*-value = 0.032, 0.011 and 0.043, respectively, Figure 3B). Some black granules were also noted in the connective tissues (Figure 4C,D).

#### 3.3.3. Hearts

**Figure 5:**
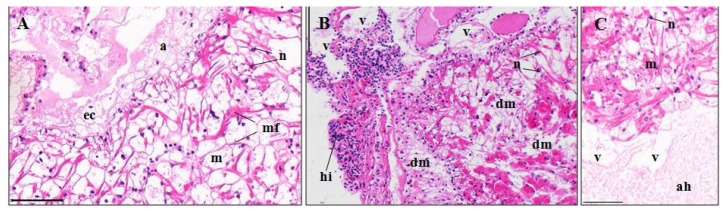
Heart structure of *P. clarkii* following 96 h exposure to a control solution (**A**) and 0.24 mg/L pymetrozine (**B**,**C**). Control hearts with normal structures of myocardium (m), adventitia (a) and multinucleated and branched myocardial fibers (mf). Note the netlike adventitia with cytoplasm-lacking and eccentrically located nuclei of the epithelial cells. ((**A**), 200×). ec, epithelial cell. Damaged hearts showing hemocytic infiltration (hi), degeneration of the myocardial fibers (dm) ((**B**), 200×), epithelial hyperplasia of the adventitia (ah) ((**C**), 200×), and vacuolation (v) (**B,C**). m, myocardium; n, nuclei; H&E stain, scale bars = 100 μm.

A normal myocardium of *P. clarkii* is composed of multinucleated and branched myocardial cells and an adventitia (epicardium) (Figure 5A). The adventitia consists of several layers of varisized and shapeless epithelial cells, which had no cytoplasm and eccentrically located nuclei, and these give the adventitia a net-like structure (Figure 5A). Following the 96-h exposure to pymetrozine, hemocytic infiltration (Figure 5B), vacuolization and hyperplasia (Figure 5C) appeared, with percentages of 2.58 ± 2.36%, 8.55 ± 1.02% and 7.74 ± 1.33%, respectively, which were significantly higher than those in the control group (*p*-value = 0.002, 0.002 and 0.004, respectively, Figure 3C). Some myocardial fibers showed swelling, fracture and lysis as well (Figure 5B).

#### 3.3.4. Stomachs

**Figure 6:**
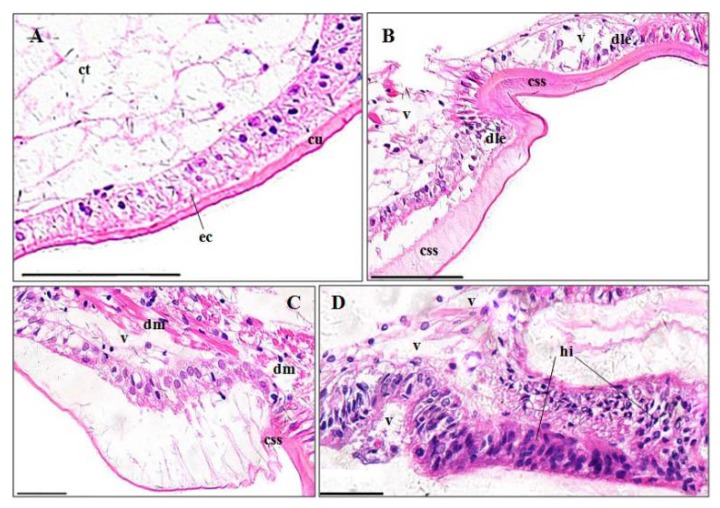
Stomach structure of *P. clarkii* following 96 h exposure to a control solution (**A**) and 0.24 mg/L pymetrozine (**B**–**D**). Control stomachs showed normal cuticula (cu), epithelial cells (ec) and connective tissues (ct) with clear and uniform structure ((**A**), 200×). Damaged stomachs showing swelling and segmentation of the cuticula (css) ((**B**), 200×; (**C**), 400×), disorganization and lysis of epithelial cells (dle) (**B**), vacuolization (v) ((**B**–**D**), 400×), muscle destruction (dm) (**C**), and hemocytic infiltration (hi) (**D**). H&E stain, scale bars = 100 μm.

A normal stomach consists of a cuticula, single epithelial layer with simple columnar epithelial cells, and a thick connective tissue layer, from outer to inner (Figure 6A). The pymetrozine-treated stomachs exhibited significantly severe cuticula swelling and segmentation (80.82 ± 8.16%, *p* < 0.01) (Figure 6B,C), vacuolization (17.77 ± 5.23%, *p* < 0.05) (Figure 6B,C), muscle fragmentation (6.98 ± 1.44%, *p* < 0.05) (Figure 6C) and hemocytic infiltration (5.24 ± 0.65%, *p* < 0.05) (Figure 6D) (Figure 3D). Epithelial cell destruction and lysis, and detachment from the cuticula were also found (Figure 5B).

#### 3.3.5. Midguts

**Figure 7:**
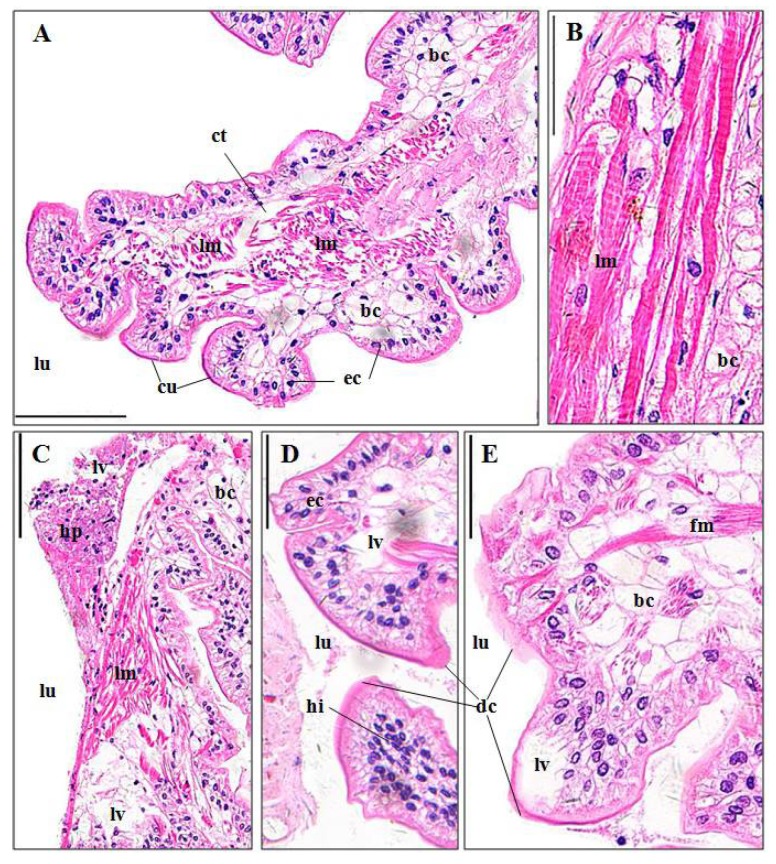
Midgut structure of *P. clarkii* following 96 h exposure to a control solution (**A**,**B**) and 0.24 mg/L pymetrozine (**C**–**E**). Control midguts with intact structures of cuticula (cu), organized epithelial cells (ec), bladder cells (bc) and longitudinal muscles (lm) ((**A**), 200×; (**B**), 400×). ct: connective tissues. Damaged midguts showing tissue hyperplasia (hp) ((**C**), 200×), hemocytic infiltration (hi) ((**D**), 200×), fracture of some muscles (fm) ((**E**), 400×), lysis and vacuolation (lv) (**C**–**E**), and degeneration and vagueness of the cuticula (dc) (**D,E**). ec, epithelial cell; bc: bladder cell; lu, lumen. H&E stain, scale bars = 100 μm.

The midgut of *P. clarkii* contains several longitudinal ridges which are composed of simple columnar epithelia and subepithelial connective tissues (Figure 7A). Cytoplasm of the epithelial cells is fibrous, and the connective tissues consist of longitudinal muscles and numerous bladder cells which with vacuoles and peripheral ovoid nuclei (Figure 7A,B). The control midguts exhibited intact and clear structure (Figure 7A,B), while in midguts after pymetrozine exposure, significantly higher percentages of hyperplasia (7.45 ± 1.44%; Figure 7C), tissue lysis and vacuolation (10.98 ± 0.84%; Figure 7C–E), hemocytic infiltration (6.74 ± 1.68%; Figure 7D), and muscle fracture (13.67 ± 5.65%; Figure 7E) appeared than those in the control midguts (*p*-value = 0.033, 0.001, 0.041 and 0.001, respectively; Figure 3E). Apparent cuticula degeneration and vagueness was also observed (Figure 7D,E).

#### 3.3.6. Abdominal Muscles

**Figure 8:**
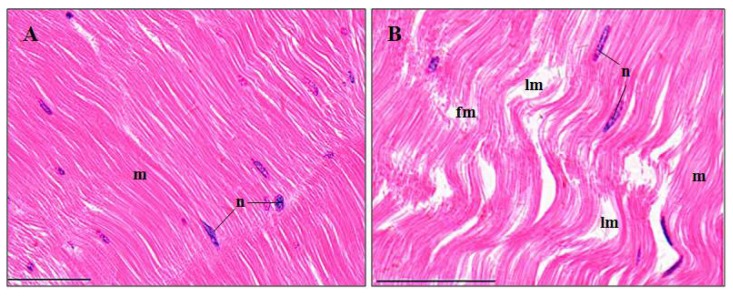
Abdominal muscle structure of *P. clarkii* following 96 h exposure to a control solution and 0.24 mg/L pymetrozine. Control muscles with ordered myofibers (m) and oval nuclei (n) (**A**). Damaged muscles showing myofiber fracture (fm) and lysis (lm) (**B**). m, myofiber; n, nucleus. H&E stain, 400×, scale bars = 100 μm.

The structures of the control abdominal muscles were generally compact and damage-free (Figure 8A). 14.09 ± 2.46% of the pymetrozine-exposed muscles exhibited myofiber fracture and lysis (Figure 8B), which was significantly higher than that of the control group (*p* < 0.05, Figure 3F).

## 4. Discussion

### 4.1. Lethal Effects of Pymetrozine

The estimated LC_50_ of pymetrozine was 0.48 mg/L, indicating a high toxicity (0.1–1 mg/L [24]) to juvenile *P. clarkii*. Unexpectedly, pymetrozine exhibited a much lower LC_50_ to *P. clarkii* than other organisms in Table 1, suggesting that *P. clarkii* is much more sensitive and vulnerable to pymetrozine than many other species. This may be due to the certain physiological similarities between crustacea and insects [16]. Pymetrozine acts on insects in a unique way interfering in the neuroregulation or nerve-muscle interaction of feeding behavior (blockage of stylet penetration), resulting in starvation to death [13,25]. However, the mechanisms of pymetrozine on sucking insects are still largely unknown.

### 4.2. Behavioral Effects of Pymetrozine

When an organism is exposed to a contaminant, it either remains unaware of the environmental change, or chemosensory perception initiates a suite of behavioral responses, such as avoidance, locomotion, feeding and mating [26]. Irritation at the start of the exposure are consistent with the universal behavioral responses of crayfish to ethion [3], thiamethoxam [16], 2,4-D [27], and etofenprox [28]. Hyperactivity may accelerate crayfish to death by enhancing agonism [16] and oxygen consumption [3,29]. The subsequent changes, such as slow movement, equilibrium loss and lethargy, could increase the vulnerability to predation in the environment, and affect the survival rate, feeding and growth of the survivals [1,30].

### 4.3. Histopathological Effects of Pymetrozine

#### 4.3.1. Gill

Gill has been demonstrated to be a major target of water-borne contaminants and the first organ showing histological changes [3]. For example, gill was identified as the most severely affected and the first organ to show pathology in pentachlorophenol-treated *Palaemonetes pugio* [31]. At a concentration of 0.24 mg/L, pymetrozine caused significant degeneration in cuticula and epithelial cells, and hemocytic infiltration, which are similar to the alterations in crustacean gills caused by trichlor, chlorpyrifos, ethion, and etofenprox [3,23,28,32]. Gill is the major organ for respiration, osmotic and ionic regulation, so pymetrozine may disrupt respiratory and osmoregulatory functions of crayfish, ultimately resulting in mortality. This has been exampled by studies on the effects of fenitrothion and trichlorfon in crustaceans [32,33].

#### 4.3.2. Perigastric Organ

As a major gland for digestion, absorption, secretion, excretion and detoxification in crustacea, perigastric organ is sensitive to pesticides and other water-borne contaminants [34,35]. Tubule lumen atrophy, lumen dilatation, vacuolation, and epithelial cell lysis in perigastric organ of crayfish in this study were consistent with other studies [3,5,6,23]. These pathological changes could, due to accumulation of pymetrozine or its degradation products in perigastric organs, lead to increased activity of the lysosomal enzymes which damages the cell organelles [3].

#### 4.3.3. Heart

Cardiotoxicity of *P. clarkii* has been previously reported after mixture of bensulfuron-methyl and acetochlor, chlorpyrifos, and recombinant VP28 protein exposure [6,23,36], including the lesions of cardiac muscle fiber, epithelial hyperplasia, hemocytic infiltration, vacuolization, and myocardial edema. Except for myocardial edema, other symptoms were also detected in the present study. Structural abnormality of heart could lead to cardiac malfunctions. For example, toxicant-induced heart malformations and reduction in heartbeat rate were observed in different fish species [37,38,39,40].

#### 4.3.4. Stomach

Pymetrozine induced similar lesions in stomachs of crayfish *Penaeus monodon* and *P. clarkii*, exposed to mixture of bensulfuron-methyl and acetochlor [6] and triazophos [13].

#### 4.3.5. Midgut

Toxicant-induced intestine deterioration has been observed in different crustacean species like *P. clarkii* [6], *P. pugi* [31], *P. monodon* [41], *Paratelphusa masoniana* [42]. In this study, midgut lesions are similar to the results of above-mentioned studies.

#### 4.3.6. Abdominal Muscle

Muscle is usually not a target organ and accumulates a relatively low amount of toxicants, due to its low-fat content [43,44]. Carapace of crustaceans may also help to prevent abdominal muscle to be damaged, which is suggested by higher content of fluoride, ethion and its degradation products in carapace than in abdominal muscle [3,45]. However, when detoxification systems are saturated, abdominal muscle may still suffer damages [44]. The pymetrozine-exposed muscles exhibited distinct myofiber fracture and lysis, which were also detected in *P. clarkii* treated with chlorpyrifos, and a mixture of bensulfuron-methyl and acetochlor [3,6].

### 4.4. Pymetrozine Safety Evaluation for RCIS

According to the estimation by Yu et al. [6], the water volume of a typical RCIS in China is over 10^8^ L/ha. Given the recommended dose of pymetrozine on rice is under 600 g a.i./ha [6,21], the practical concentration of pymetrozine in RCIS water is estimated less than 6 μg/L, which is far lower than the calculated MAC (0.106 mg/L). In this sense, a proper application of pymetrozine in RCIS is unlikely to induce mortality of crayfish. However, the MAC was calculated based on only mortality data from acute tests and the potential impact of chronic exposure is still unknown. In addition, the actual environmental concentrations of pymetrozine in RCIS could be elevated or even higher than the recommended dose due to the possible misconduct (over-dosing) of pymetrozine application, long-term application of pymetrozine, accumulation of pymetrozine in particles and sediment, shallowed water in RCIS etc., implying the concerns and uncertainties in the safety of pymetrozine application in RCIS.

## 5. Conclusions

The present study revealed that pymetrozine was highly toxic to juvenile *P. clarkii*, with the 96 h LC_50_ and maximum allowable concentration of 0.479 and 0.106 mg/L, respectively. Pymetrozine caused behavioral abnormalities at lethal and sublethal concentrations (0.01–1.1 mg/L), and significant pathological changes in gills, perigastric organs, hearts, stomachs, midguts and abdominal muscles at 0.24 mg/L. The results, to our knowledge, are the first to provide a comprehensive toxicity dataset incorporating both lethal and sublethal measurements to better understand the impact of pymetrozine to crayfish, and call for further long-term investigations on the environmental fate (e.g., degradation, bioaccumulation, and transformation) and chronic effects of pymetrozine.

## Figures and Tables

**Table 1:** Estimated acute toxicity of pymetrozine to some ascertained aquatic and ricefield-relative organisms.

Organism	LC_50_/EC_50_ (mg/L)	US EPA Toxicity Category	Reference
*Oncorhynchus mykiss*	>128 (96 h LC_50_)	Practically non	[17]
*Lepomis macrochirus*	>134 (96 h LC_50_)	Practically non	[17]
*Cyprinodon variegatus*	>117 (96 h LC_50_)	Practically non	[17]
*Brachydanio rerio*	119.84 (96 h LC_50_)	Practically non	[18]
*Carassais auratus gibebio*	387 (96 h LC_50_)	Practically non	[19]
*Lemna gibba*	>109 (EC_50_)	Slightly	[17]
*Daphnia magna*	87 (48 h EC_50_)	Slightly	[17]
*Kirchneria subcapitata*	17 (EC_50_)	Slightly	[17]
*Hyposoter didymator*	–	Harmless	[20]
*Trichogramma chilonis*	0.96 (48 h LC_50_)	Slightly–moderately	[21]

**Table 2:** Regression equations, LC_50_ values, and 95% confidence limits of pymetrozine to juvenile *P. clarkii* at 24, 48, 72 and 96 h.

Time (h)	Regression Equation	R^2^	LC_50_ (mg/L)	95% Confidence Limits (mg/L)
24	*P* = -0.050 + 3.472*C*	0.927	1.034	0.875–1.392
48	*P* = 0.316 + 2.254*C*	0.904	0.724	0.588–0.942
72	*P* = 0.650 + 2.515*C*	0.942	0.551	0.452–0.671
96	*P* = 0.880 + 2.753*C*	0.973	0.479	0.393–0.571

In the regression equation, R^2^, *P* and *C* are the regression coefficient, probability unit of mortality, and logarithm of pymetrozine concentration, respectively.
